# Supplementary material for: The Discovery of Oropharyngeal Microbiota with Inhibitory Activity against Pathogenic Neisseria gonorrhoeae and Neisseria meningitidis: An In Vitro Study of Clinical Isolates
Source: Microorganisms. 2022 Dec 16;10(12):2497. doi: 10.3390/microorganisms10122497 (PMC9787740; doi:10.3390/microorganisms10122497)
Supplement: Supplementary file 1 [file microorganisms-10-02497-s001.zip › microorganisms-2050469-supplementary.pdf]

Supplementary Materials

Table S1. Inhibitory Isolates (62 isolates, 14 species, 2 genera).

ID	Sample Type	Pharyngeal Gonorrhoea	No of Isolates	Inhibitory to *N. Gonorrhoeae*	Inhibitory to *N. Meningitidis*	MALDI-TOF ID
7. M0	Baseline	Negative	3	Yes	Yes	*Streptococcus pyogenes*
8. M0	Baseline	Negative	2	Yes	Yes	*Streptococcus cristatus*
8. M0	Baseline	Negative	3	Yes	No	*Streptococcus oralis*
10. M0	Baseline	Negative	2	Yes	No	*Streptococcus parasanguinis*
12. M0	Baseline	Negative	1	Yes	No	*Streptococcus salivarius*
13. M0	Baseline	Negative	1	Yes	Yes	*Streptococcus mitis*
13. M0	Baseline	Negative	2	Yes	Yes	*Streptococcus parasanguinis*
13. M0	Baseline	Negative	3	Yes	Yes	*Streptococcus salivarius*
13. M0	Baseline	Negative	4	Yes	Yes	*Streptococcus mitis*
15. M0	Baseline	Negative	1	Yes	Yes	*Streptococcus dysgalactiae*
15. M0	Baseline	Negative	2	Yes	No	*Streptococcus dysgalactiae*
15. M0	Baseline	Negative	3	Yes	No	*Streptococcus parasanguinis*
44. M0	Baseline	Negative	2	Yes	No	*Streptococcus cristatus*
45. M0	Baseline	Negative	1	Yes	No	*Streptococcus cristatus*
51. M0	Baseline	Negative	1	Yes	Yes	*Streptococcus sanguinis*
51. M0	Baseline	Negative	3	No	Yes	*Streptococcus sanguinis*
51. M0	Baseline	Negative	4	Yes	Yes	*Streptococcus sanguinis*
51. M0	Baseline	Negative	5	No	Yes	*Streptococcus salivarius*
51. M0	Baseline	Negative	6	No	Yes	*Streptococcus sanguinis*
51. M0	Baseline	Negative	7	No	Yes	*Rothia mucilaginosa*
53. M0	Baseline	Negative	2	Yes	Yes	*Streptococcus oralis*
56. M0	Baseline	Negative	1	Yes	No	*Streptococcus mitis*
56. M0	Baseline	Negative	2	Yes	Yes	*Streptococcus mitis*
36. M3	Treatment	Negative	1	Yes	No	*Streptococcus parasanguinis*
36. M3	Treatment	Negative	2	Yes	No	*Streptococcus sanguinis*
36. M3	Treatment	Negative	3	No	Yes	*Streptococcus parasanguinis*
42. M3	Treatment	Negative	1	Yes	Yes	*Streptococcus sanguinis*
3. M3	Treatment	Negative	1	Yes	No	*Streptococcus vestibularis*
43. M3	Treatment	Negative	1	Yes	Yes	*Streptococcus peroris*
13. M3	Treatment	Negative	1	Yes	Yes	*Streptococcus parasanguinis*
13. M3	Treatment	Negative	2	Yes	Yes	*Streptococcus oralis*
16. M3	Treatment	Negative	1	Yes	Yes	*Streptococcus parasanguinis*
16. M3	Treatment	Negative	2	Yes	Yes	*Streptococcus mitis*
16. M3	Treatment	Negative	3	Yes	Yes	*Streptococcus vestibularis*

15. M3	Treatment	Negative	1	Yes	Yes	*Streptococcus parasanguinis*
15. M3	Treatment	Negative	2	Yes	Yes	*Streptococcus parasanguinis*
47. M3	Treatment	Negative	1	Yes	Yes	*Streptococcus gordonii*
47. M3	Treatment	Negative	4	Yes	Yes	*Streptococcus parasanguinis*
24. M3	Treatment	Negative	1	Yes	Yes	*Streptococcus oralis*
29. M3	Treatment	Positive	1	Yes	Yes	*Streptococcus parasanguinis*
29. M3	Treatment	Positive	2	Yes	Yes	*Streptococcus parasanguinis*
12. M6	Treatment	Negative	1	Yes	No	*Streptococcus cristatus*
23. M6	Treatment	Negative	1	Yes	No	*Streptococcus salivarius*
23. M6	Treatment	Negative	2	Yes	No	*Streptococcus oralis*
23. M6	Treatment	Negative	3	Yes	No	*Streptococcus sanguinis*
31. M6	Treatment	Negative	1	Yes	No	*Streptococcus sanguinis*
31. M6	Treatment	Negative	2	Yes	No	*Streptococcus sanguinis*
31. M6	Treatment	Negative	3	Yes	No	*Streptococcus sanguinis*
59. M3	Treatment	Negative	2	No	Yes	*Streptococcus mitis*
59. M3	Treatment	Negative	3	No	Yes	*Streptococcus dysgalactiae*
60. M3	Treatment	Negative	2	Yes	No	*Streptococcus infantis*
28. M6	Treatment	Negative	2	Yes	Yes	*Streptococcus parasanguinis*
28. M6	Treatment	Negative	3	No	Yes	*Streptococcus parasanguinis*
28. M6	Treatment	Negative	5	Yes	Yes	*Streptococcus pyogenes*
49. M6	Treatment	Positive	1	Yes	No	*Streptococcus pyogenes*
49. M6	Treatment	Positive	2	Yes	Yes	*Streptococcus pyogenes*
53. M6	Treatment	Negative	1	Yes	Yes	*Streptococcus pyogenes*
53. M6	Treatment	Negative	2	Yes	Yes	*Streptococcus pyogenes*
57. M6	Treatment	Negative	1	Yes	Yes	*Rothia dentocariosa*
57. M6	Treatment	Negative	4	Yes	No	*Streptococcus mitis*
57. M6	Treatment	Negative	9	Yes	Yes	*Rothia dentocariosa*
64. M6	Treatment	Postive	1	Yes	Yes	*Streptococcus mitis*
